# Developing a Reliable Service System of Charity Donation During the Covid-19 Outbreak

**DOI:** 10.1109/ACCESS.2020.3017654

**Published:** 2020-08-18

**Authors:** Hanyang Wu, Xianchen Zhu

**Affiliations:** 1 School of Economics and ManagementNanjing University of Science and Technology 12436 Nanjing 210094 China; 2 School of Economics and ManagementJilin Institute of Chemical Technology 71106 Jilin 132022 China

**Keywords:** Blockchain technology, the covid-19 outbreak, charity donation, service system, reliability

## Abstract

Drawing upon the functional characteristics of blockchain technology, this article envisages the feasibility and reliability of developing a charity donation service system loaded onto blockchain in response to the complex service demands encountered by charity operators due to the Covid-19 epidemic. With blockchain technology’s support as the underlying data book, this article focuses on the practical issues of charity donation fund and material allocation, as well as information release and sharing, charity donation organization, and organization self-management. The paper thereby discusses the key technologies in terms of overall structure design, specific service sector, and functional design of the donation service system and further summarizes the operational mechanism of the system as combined with the needs of help-seeking, receiving, and management users. It is argued that all the above proposals have the potential to alleviate the trust crisis of charity services in China in view of low transparency. The paper expects to provide a useful reference for charity business innovation propelled by blockchain technology.

## I. Introduction

Resisting major disasters has become a common global topic, and strengthening the governance of them is an essential field of charity. In the past decade, there have been 3751 major natural disasters in the world, with a total population of 2 billion and a total loss of 1658 billion US dollars. All countries attach importance to strengthening close cooperation between the public and private sectors, various non-governmental organizations and scientific research institutions, and have formed a multi-party cooperation mechanism for disaster relief and disaster reduction. With its strong mobilization, independence, and professionalism, a charity donation can quickly organize work on disaster relief, which is an essential part of social forces in such a situation.

With the outbreak of the Novel Coronavirus (Covid-19) epidemic all over the world, it has made a significant impact on people’s social production and daily life, and post-epidemic assistance is particularly urgent. Wuhan, the capital of Hubei province, was the hardest-hit city affected by the Covid-19 in China, and the city in lockdown triggered a shortage of living and epidemic prevention materials. At that time, charity donations from all over the world rushed into Wuhan through various channels. Whereas Wuhan Red Cross actively carried out assistance work, negative news regarding its arrangements also appeared ceaselessly, such as the unfair distribution and untimely distribution of donation materials and inefficient financial settlement over the Ministry of Finance [1]. All the negative news resulted in a massive dilemma of charity donations.

Public welfare crisis in China has a long history, and there are frequent events involved in charity donations, for example, Wenchuan earthquake in 2008 [2], “Guo Meimei incident” [3], a scandal related to the financial problems of the Red Cross, and Suntech donation fraud [4]. Promoting public participation in charity donations has become an urgent social problem. With the rapid, widespread and long development cycle, the immediate demand and reasonable allocation of funds and materials have become a significant problem faced by the global fight against the epidemic. Blockchain technology ensures the credibility of the process through the Peer-to Peer (P2P) network, consensus mechanism, incentive mechanism, and smart contract. The development of a charity donation service system can solve the “trust crisis”. It can establish an efficient connection between charitable organizations and beneficiaries, can respond quickly and form a more open and transparent trust mechanism, and can solve the unprecedented problems brought about by the massive demand for donation in the new epidemic.

Under the outbreak of Covid-19, the information of charity donation is asymmetric. With the purpose of preventing this situation, the goal of our research is to establish a donation service system, where system is guaranteed by the blockchain-based technology. We believe that this can eliminate asymmetry, and effectively deal with asymmetry and bursts. This article follows a case-study design, with in-depth theoretical analysis of blockchain technology. The characteristics of blockchain could be very helpful in establishing such donation service system. In the remainder of the paper, we firstly review the literature on the blockchain technology, as well as past research on charitable behaviors. The third part begins by laying out the charity donation service system from the perspective of demand. The fourth part presents the design of charity donation service system, focusing on the three key themes that have been identified in analysis. The fifth part explains the implementation of the service system, and finally the conclusion gives a brief summary.

## II. Blockchain Technology and Application of Charity Donation Industry

Blockchain technology is to provide decentralized ledgers whose distribution is in the form of cryptography in chronological order [5]. Each block is a linked data structure in the form of a linked list. The data in the block cannot be tampered but can be verified in the system, and they can be stored safely in a sequential relationship. Data are distributed through an extensive, distributed, and incorruptible network of computers, allowing us to interact with stored data timely, without direct intermediaries and reliance on conventional, proprietary, closed, and hard-to-control IT infrastructures [6]. Blockchain provides a new independent, tamper-proof, and transparent platform to securely store, transmit and process sensitive and valuable data [7], [8].

In 2008, Satoshi Nakamoto, the founder of Bitcoin, established the Bitcoin system. In 2009, blockchain technology as the underlying technology of Bitcoin was firstly applied [9]. With the development of the Bitcoin market, blockchain technology has further grown and gradually attracted the attention of the industry, governments, and scholars [10].

In 2019, China’s central government specially organized blockchain technology training on development status and trend learning. As an essential breakthrough in core technology and innovation, blockchain technology has strengthened and developed blockchain in many fields, such as digital finance, Internet of goods, intelligent manufacturing, supply chain management, digital asset trading, etc. [11]–[14]. At that time, each provincial administrative region immediately issued relevant industrial policies to promote industrial development in various forms. As a cutting-edge field of computer technology, blockchain technology is expected to become a significant innovation which will change the development of human society after the revolution of steam technology, power technology and information technology [15]–[17].

The blockchain research mainly focused on both technology itself and its industrial applications. In the technological field, a consensus is reached, that is, a series of ordered data blocks are generated by related technologies, such as mathematical algorithms, cryptography, computer science, etc. to form a reliable database [18], [19]. The system incorporates multiple characteristics, including sequentiality, decentralization, de-reliability, tamper-proof, and traceability; and all is to realize data verifiability and to secure storage. Reference Zhang *et al.* focused on user access control and data traceability of user published data information [21]. Hasan *et al.* summarized the problems and challenges of security traceability at first. He pointed out that a single traceability record is to be ensured, and the order of the record owner is not modified, both of which are based on the security traceability model [22]. Blockchains allow us to have a distributed Peer-to-Peer network where non-trusting members can interact with each other without a trusted intermediary, in a correct manner [23].

Due to its technological characteristics, the application of blockchain technology in charity donation and related industries has received extensive attention. Reference Liu *et al.* [24] summarized the opportunities, benefits, and challenges of incorporating blockchain in different industrial applications such as the financial industry, healthcare industry, logistics industry, manufacturing industry, energy industry, agriculture and food industry, robotics industry [25]. Lu *et al.* analyzed opportunities, challenges, and risks and development trends of blockchain in the oil and gas industry [26]. Qi-Feng *et al.* summarized a sort of architecture based on the blockchain system for the mainstream blockchain development framework [27], which makes it more perfect for constructing a charity donation system based on the blockchain technology. Jia & Deng held that blockchain technology could establish new information sharing and trust mechanisms to improve the operation efficiency of the social assistance system [28]. Jia and Hai-Feng believed that blockchain technology could solve the problem of the credit formula system in China’s charity and improve the credibility of charitable organizations [29]. Scholars commenced on the design and implementation of the charity donation system based on blockchain technology, and thus formed cases on the industry application [30], [31]. Nor *et al.* set up a fundraising system for post-disaster rescue, which can be used for post-disaster fundraising and donation based on blockchain technology [32]. Danushka Jayasinghe established a charity donation platform based on bitcoin, which realized a safe, fast, and convenient transaction mode. Mainly the platform can be applied for solving the problems when there was no network after the disaster so that the donation could be completed by mobile GSM network [33]. Jakobsson *et al.* proposed a set of consensus algorithm for blockchain application to solve the trust problems between machines, and to solve the issues of multi-center credit mechanism through technical means, which is conducive to the development of charity donation system based on alliance chain [34]. Bo Zhang believed that blockchain of bitcoin could deal with Byzantine failures, realized the connection of historical information through workload proof, and formed the primary chain for solving the information traceability of charity donation materials [35]. Maesa *et al.* provided a resource access scheme of blockchain, which can help users obtain access to resources in the system in time and prevent fraudsters from no executing system rules through distributed audit [36]. Hui-Juan and Wen-Yon pointed out that the blockchain trust mechanism could reduce transaction costs, promote the trust mechanism of both sides of the transaction in specific scenarios, and thus improve the success rate of transactions. Notably, it can realize point-to-point direct transactions, reduce the barriers of information asymmetry, and help to provide mode selection for charity donation financial business. [37]

We performed the literature review to demonstrate the advantages and possibilities of blockchain technology applied in the donation service system. Based on a considerable amount of literature proposed in the paper, we outlined several contributions to the research on charity donations. Firstly, we take the Covid-19 as an example to discuss how the charity donation system is designed, applied, operated and implemented in China, this is of great, pragmatic and practical significance. Secondly, Blockchain technology, with characteristics of traceability, transparency, security and privacy, could contribute to reorganize the production, transaction and management of the donation behaviors, we affirm that this technology provides to the charity donation system full control, security and responsibility over traditional charitable behaviors.

## III. Requirements for the Construction of the Charity Donation Service System During The Covid-19 Outbreak

### A. The Current Situation of Charity Donation for New Epidemic Situation

The participation of charitable organizations mainly focuses on the provision of materials and funds, which can quickly grasp the situation of disasters and mobilize resources based on rapid response intelligence systems when disasters occur [38]. China’s charity donation has been developing unprecedentedly since the 1980s. It has set a primary direction of “fundraising, medical and health care, and traffic reconstruction, disaster relief linkage,” and of relying on new technologies and new platforms to enhance the capacity, efficiency, and response speed of disaster relief [39]. After the Covid-19 outbreak, the government and the charity forces cooperated to build a local government “one case, three systems” major disaster management system [40] according to the unstructured disaster reduction theory [41]. Various social organizations and the government established multiple collaborative networks, which were large-scale, multi-party participation, flexible, and efficient. Within the first week of the Covid-19 outbreak, according to the data released by China Charity Federation, the national charity donations reached 10.138 billion yuan, which did not include the converted value of some material donations. The sustained development and global spread of the Covid-19 have brought unprecedented public concern and social sensitivity to charity donations and faced more significant challenges.

### B. Demand Characteristics During the Covid-19 Outbreak

The Covid-19 epidemic reflects the essential characteristics of major disasters, which are usually sudden, destructive, and uncertain. After the occurrence of major disasters, there are always sudden changes in demand and supply in the short term, which are mainly reflected in the following aspects. Firstly, the need for epidemic prevention materials is increasing rapidly, and the scale is unprecedented. The need for protective clothing, masks, thermometers, and disinfectants affects the global production and supply of epidemic prevention materials; secondly, the demand is urgent. Major disasters have their uncertainties, and the reserves of disaster relief materials are often lack of planning. Usually, the emergency of major disasters makes it challenging to supply disaster relief materials in a short time. The supply of masks in the early stage of the Covid-19 was affected by many factors, such as the production cycle. It took China one month to increase the production capacity of masks from 20 million per day to 1.8 billion per day; thirdly, the relationship between supply and demand is complicated. On the one hand, it is a complex demand for help, including not only epidemic prevention materials but also daily assistance such as clothing, food, housing, transportation, and medical care brought about by the epidemic. On the other hand, it is necessary to establish a sound allocation system for a large number of donated funds and materials to achieve the expected goals of charity donations.

### C. Demands of Service System Construction

#### 1) Information Release and Sharing

The release of major disaster information requires timeliness, authenticity, accuracy, rapid transmission, and useful feedback. The sudden outbreak of the Covid-19 has brought about the shortage of epidemic prevention materials, soaring prices, and hoarding. From families and communities to social groups and local governments, they have put forward complex rescue demands. At the same time, the help-seeking information is rapidly transmitted, amplified, and even distorted through media attention or other transmission channels. At present, donation awareness and charity participation from Chinese people are not strong. As the representative of the leading charitable donor, Chinese entrepreneurs still lack systematicness, autonomy, and balance. As legal management organizations, public welfare social organizations such as the Red Cross need to create a positive atmosphere to promote charity activities. The public needs to obtain accurate information to encourage their willingness to help. The tracking and feedback of donations reflect the effectiveness and credibility of public welfare organizations and have an impact on the enthusiasm and initiative of the public to participate. [42]

**FIGURE 1.**
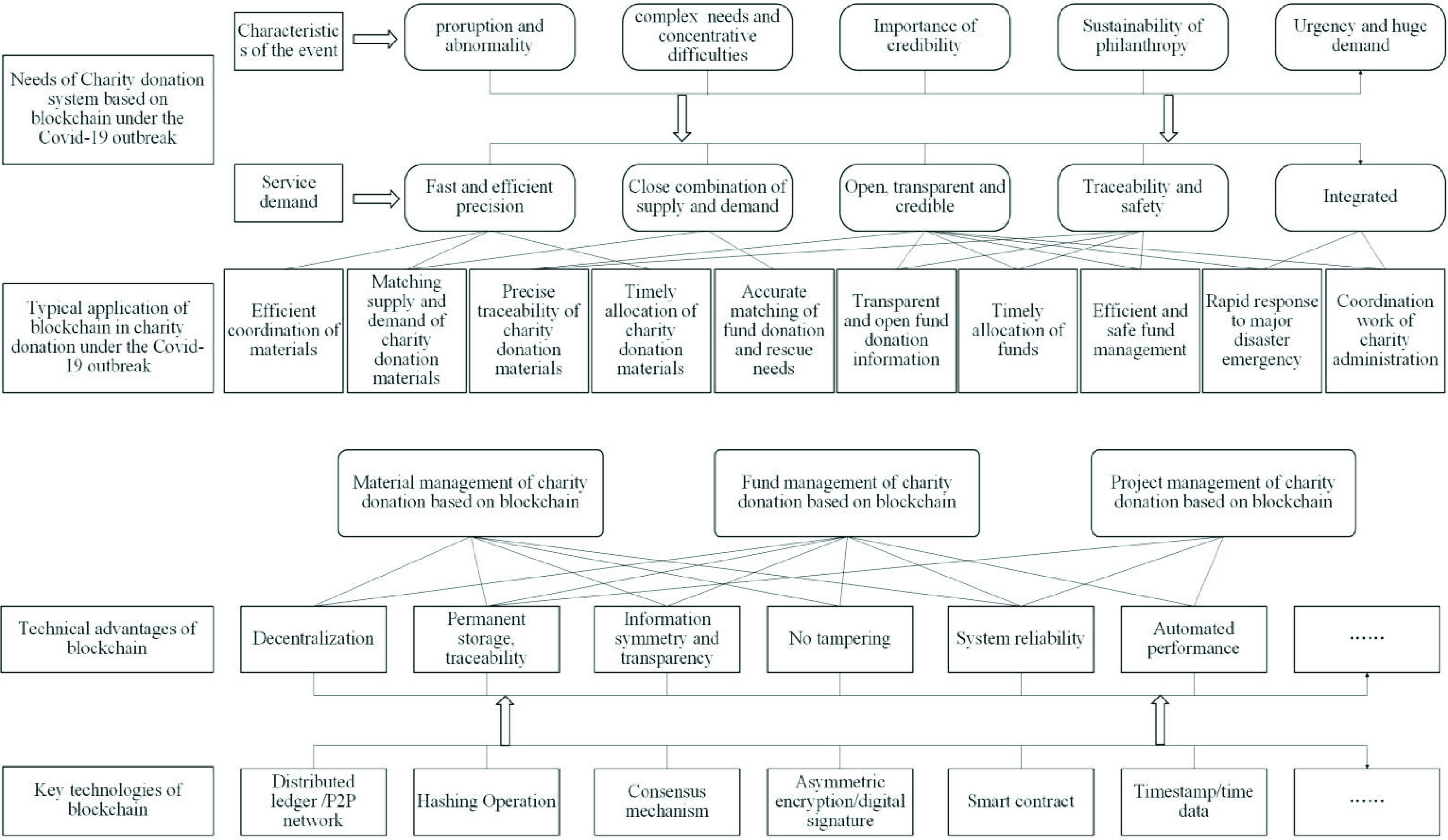
The combination mechanism of blockchain and charity donation.

#### 2) Allocation of Materials and Funds

Charity donation shows the humanitarian care from organizations and individuals. Social and human capital and religious beliefs play a critical role in determining individuals’ charity behaviors [43]. Charity donation can help the beneficiary by free donation or by giving the legal property that they have the right to dispose of [44]. After the Covid-19 outbreak, relief materials and funds increased in a blowout way, far exceeding the historical high of 45.8 billion yuan donated by individuals in the 2008 Wenchuan earthquake. Reference [45] According to the “charity law” and “public welfare donation law”, donors can either choose the Red Cross or other public welfare social organizations or non-profit organizations to donate or directly to the beneficiaries. Based on donors’ willingness and the beneficiaries’ needs, a large number of materials and donations awaited to be allocated in time. Moreover, Wuhan even faced the problem on illegally issuing masks in the city’s emergency materials reserve warehouse. As a result of Wuhan’s temporary non-acceptance of overseas donations, overseas relief materials were stranded in the local airport. They had no way to be released, which affected the practical promotion work.

#### 3) Operation Mechanism of Charity Donation

Wuhan is not alone. Despite all kinds of problems in the donation process, the popularity of non-governmental charitable organizations continues to grow, and the number of organizations and individuals involved is also increasing. According to the statistics of the Foundation Center network, as of May 30, 2019, there are 7358 foundations nationwide. Among them, there are 1612 public foundations and 5746 non-public foundations. According to the existing laws and regulations in China, the development of charity is dominated by public welfare organizations and non-profit institutions. Official or semi-official organizations receive most of the donated materials. The main channel of charity donation is an indirect donation, and the Ministry of civil affairs and the general Charity Association are the primary recipients. The uncertainty of the Covid-19 epidemic requires that the supply scale and allocation speed of donated materials and funds should be released rapidly, and the charity donation organizations, contents, methods, and operation efficiency need to be coordinated and strengthened [46]. At the same time, charity donations are not limited to materials and funds, but also includes service donation, equity donation and other new forms of donation, such as transportation services, communication services, mental health services, insurance services, etc. The diversification and innovative development of donation resources urge charitable organizations, donors, and beneficiaries to reconstruct the trust relationship among all sides of charity, earnestly perform their duties and supervise the operation of charity mechanisms.

#### 4) Internal Management of Charitable Organizations

Means of management for charitable organizations determine their development quality. China’s charitable organizations experience a relatively short development, and there are many deficiencies in internal administration, such as credit mechanism, supervision mechanism, personnel management system, and financial management system [47]. According to Foundation Transparency Index (FTI) guidebook 2019, China’s FTI is 51.34 points, which has increased in terms of information disclosure and necessary information disclosure. Still, the completion rate in terms of project information and financial information is only 37% and 41%, especially in terms of the completion of compliance indicators [48]. Donors, beneficiaries, and the public need to supervise the process of charity donation transparently. Philanthropic organizations need to solve the problems of information symmetry and credibility to develop better. The application of blockchain technology can solve the problem of efficient connection between all parties, integrate logistics, finance, performance evaluation, and other technical support, can form a positive interaction mechanism, and can establish a credible charity donation service system.

## IV. System Design of Charity Donation Service System During the Covid-19 Outbreak

### A. Feasibility Analysis

During the Covid-19 outbreak, rescue is urgent and complicated, especially the rescue information is confused, and the rescue precision is delicate. The most important advantage of choosing blockchain technology to design and develop the charity donation service system is procedural and long-term credible, to ensure that charity donation can play an expected role [49]. At present, the Bubi blockchain platform is widely used, with rich operating experience and many successful cases. Therefore, using the Bubi as the underlying blockchain of the service system can ensure the system stability and convenient docking.

In terms of equipment and technical feasibility, Ethereum has formed a well-functioning blockchain application and development platform, which is well-recognized, mature, and stable in technology [50]. There are many development frameworks and software tools on Ethereum, with complete development language and debugging tools, to reduce the demand for high-performance equipment and other hardware investment. The system is primarily based on the Web application of blockchain, which is easy to develop and to be used. Meanwhile, in terms of technology, J2EE, mainstream in the application market, is used to design the construction scheme of an enterprise-level distributed application platform, and simplifies and standardizes the development and deployment of the system, which has marked advantages in web page dynamics, commercial components, message transmission mechanism, database access, and security management. The front-end is developed by HTML and JavaScript programming language, and the back-end database is built by Java and Spring framework, both of which are relatively mature. The most noteworthy feature of the system is lightweight [51]. Mybatis is used to support SQL queries and realize an excellent persistence framework of a stored procedure and advanced mapping. Redis and Ehcache are applied to achieve the dual cache framework, which is fast, persistent, and rich in data. The Spring MVC, the most popular MVC framework, is used to realize lightweight of the demand-driven type, and, at the same time, it can adequately support the development and operation of My SOL relational database.

**TABLE 1:** Comparisons Between Blockchain Platform.1

Items	Ethereum	Hyperledger Fabric
Overview	regular blockchain platform	modularity and extensibility of blockchain platform
Application scope	specific field	finance, logistics, e-commerce, and other industries
administrative organization	Ethereum Foundation	Linux Foundation
Operation mode	unauthorized, public or private	authorized, private
Consensus mechanism	PoW	various mechanisms
Smart contract	solidity	Chain codes

Charity donation service system integrates mature software development technology, which can support the development and operation of the system well and can be most reliable to help donation services under the Covid-19 epidemic.

**FIGURE 2.**
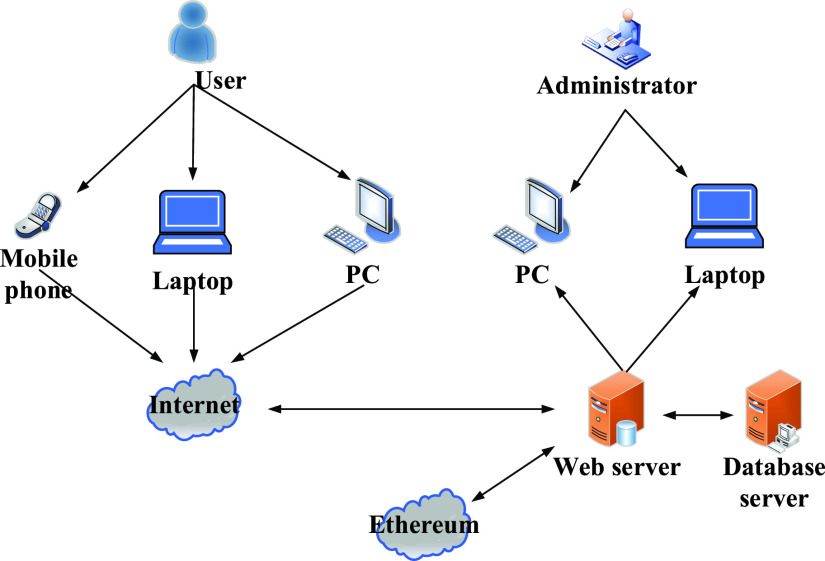
The physical framework of charity donation service system based on blockchain.

### B. Design and Operation of Service System

#### 1) Overall Framework of System

According to read-write access permission and decentralization degree, the blockchain can be divided into three types: public chain, private chain, and alliance chain [52]. In general, the higher the degree of decentralization, the higher the credibility, but the transaction speed is slower. The public chain is a highly decentralized distributed ledger, with open data, transparent transaction, stable operation without being tampered, so the public chain promotes the development of bitcoin blockchain and Ethereum smart contract [53]. However, it has the disadvantages of excessive bookkeeping steps and poor transaction timeliness. The private chain has the advantages of fast transaction speed, low transaction cost, and timeliness, but the node is not equal, and it is too centralized and easy to be controlled by the initiator. The alliance chain is more transparent and decentralized than the private ones, and members of the alliance jointly maintain the nodes. With involved authority and high credibility, at present, the Hyperledger project is the largest alliance chain in the global market, and it is the first open-source distributed ledger platform for enterprise application scenarios.

**TABLE 2:** Comparisons Among Public Chain, Private Chain and Alliance Chain

Technological factors	Public chain	Private chain	Alliance chain
Read-write access	Public	Specific individuals or groups	Authorized Individuals or groups
Bookkeeper	All subjects	Controller	Pre-selected bookkeeper
Degree of centralization	Decentralization	Centralization	Multi-centered
Trust mechanism	Proof-of-Work	Self-endorsement	Collective endorsement
Operating rate (transaction/second)	3–20	1000–100000	1000–10000
Barriers to entry	Low	Extremely high	High
Incentives	Yes	No	Selectable
Application	Digital currency, financial assets transaction	Internal use of specific transaction	Clearing and settlement systems
Typical case	Bitcoin, Ether	Ant Financial	Hyperledger

Under complex conditions with various organizations and individuals, a charity donation service system is more suitable to adopt the public chain combining with characteristics of alliance chain, which can realize decentralization and data authenticity under the premise of transaction restriction [54].

According to the needs of the Covid-19 epidemic, the charity donation service system realizes the whole process management. Decentralization can ensure that the data in the system cannot be tampered with, authorize all parties to query, receive, process, and store in time. According to the overall framework of the system, charitable organizations, donors, and beneficiaries publish information after registering as users. Then the system database obtains, stores, and processes data, and the general data and file summary information are stored in the blockchain. Under the consensus mechanism and smart contract [55], the donation project is established and operated. All parties fully mobilize the resources of producers, purchasers, banks, logistics providers, communities, and volunteers, and the clearing and settlement are more convenient. The system promotes all parties to be adequately supervised, with apparent authority, traceability, and transparency, and is suited for project evaluation.

**FIGURE 3.**
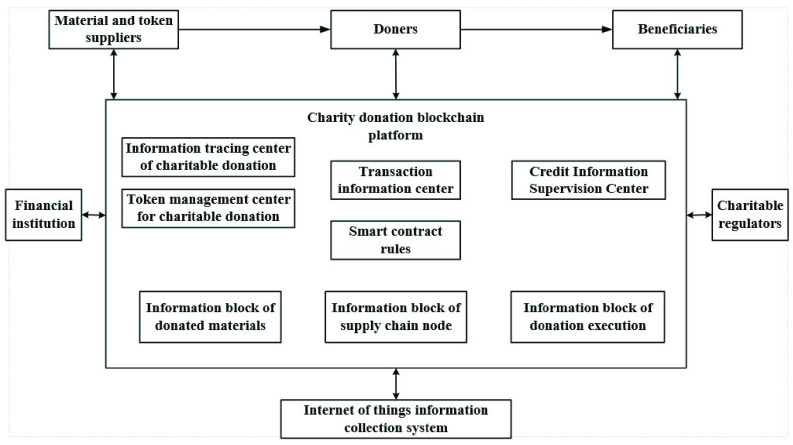
Concept model of charity donation service system based on blockchain.

#### 2) Business and Functions

Blockchain system requirements. ①initial coin offering and material donation management function. In the charity donation service system, Ethereum virtual token is used as the fund donation carrier, and information management is carried out for the logistics supply of donation materials; ②charity donation project is established. In the charity donation service system based on blockchain, the charity project corresponding to the charity donation is developed, and the fund in the project and logistics information of the charity project can be accurately recorded; ③project query functions. Either charity donors or beneficiaries can review the plans on the blockchain and track the flow of funds and materials; ④charity donation distribution function. The system can distribute the funds of charity donation to the designated project or the beneficiary’s account or keep a record of the logistics information of charity donation materials; ⑤the performance evaluation function. It mainly considers the effect of charity donation and the credibility of charity organizations, both of which make the behavior of charity donation more transparent and can solve the needs of charity donation termination, fund, and material recovery.

Business service requirements. ①information release: The scale and duration of the Covid-19 outbreak are unprecedented, covering a more extensive range of areas. Information release and access need to be timely, accurate and convenient; ②application for rescue: the beneficiaries release help information through the front end of system platform; ③online donation: it can browse online in time through the service system and select appropriate projects to donate; ④project management: it uses the service system to create, manage and end charitable projects, grasp the necessary information, donation progress, donation results, etc.; ⑤fund and material records and queries of charity donations; ⑥information disclosure of charitable management institutions, philanthropic organizations, and individuals; ⑦user management; ⑧charity project evaluation.

User management requirements. It mainly defines the scope of beneficiaries under the Covid-19 epidemic. The service system needs to locate the scenarios according to the roles of users, to meet different needs. The user categories are divided as follows: ①the charity management organization as the chief information reporter of major disasters; ②the beneficiary is the group in urgent need of help, using the system for information release and directional donation; ③the donor including charity organizations and charity individuals, can timely obtain charity project information, and carry out charity donation and track charity donation information; ④the charity project manager in charge of creating, reviewing, releasing, monitoring and achieving the project; ⑤information administrator is responsible for publishing and managing charity donation information; ⑥donation administrator is mainly charge of funds and materials, including account management, money-transfer, material allocation and material distribution, etc.; ⑦evaluation and management of the organization is able to evaluate the project and ensure the transparent management; ⑧system administrator in charge of account, business, software system, App, website and other management.

### C. Operation of Service System

The service system can promote the information exchange and information sharing among users, establish and play a role through the help-seekers, project administrators, donors, financial administrators, system administrators and other business modules at all levels, so as to make the charity donation projects run efficiently, get adequate supervision, and give full play to the rescue role in time.

#### 1) Beneficiary Module and its Operation

① the beneficiary registers and login in, including basic data entry, identity information verification, and video and audio collection; ②information release: including the present situation of the Covid-19 epidemic, needs and methods of support; ③information maintenance means maintain and update the information of system users, including personal accounts, contact information, etc. The central part of maintenance is the update of the change and status of the disaster, and update on the latest demand of donated materials and funds, etc.; ④progress query includes the set-up, approval, operation and browsing status of charity donors; ⑤project completion is responsible for the input of donated materials and funds, the feedback on the progress of relief, and the end of the project; ⑥ information feedback includes relief progress, effectiveness, and evaluation to fulfill the needs of a systematic assessment.

#### 2) Donors Module and its Operation

① The donor registers and login in, including basic data entry, identity information verification, and video and audio collection. ②Information query: The users of charity donation log in to the system to learn the latest status of the epidemic and the needs of beneficiaries, and to track the changes in disasters and requirements. ③Fund donation: The donation on the blockchain is carried out through a virtual token. Donors choose charity projects and recharge their accounts. ④Material donation: For donation, in the form of materials, such as masks, protective clothing, protective glasses, etc., the application of blockchain technology focuses on the management of the whole process of procurement, transportation, deployment, and distribution. In the system, it shows that purchase completed, purchase receipt, and purchase recorded. ⑤Initiating and searching for projects. Donors can browse the latest charity donation projects on websites or APP or create new donation projects according to needs and willingness, and users can browse project information and facilitate project selection. ⑥Donation operation: donors finally confirm the use of funds and materials and authorize final transformation so that the system can timely transfer donors’ funds to the designated beneficiary’s account after donors’ selection. Materials are selected to donate and allocated timely to beneficiaries after they arrive in the disaster area. A copy of the information will be sent to the designated user or application administrator for approval. Then the system will contact the recipient to apply for use. ⑦Historical record query: the donors can query the relevant charity donation records for statistical analysis, such as donation amount, donation time. Especially in the material donation and management business, charity donation users pay more attention to the use of materials. A charitable crisis is often caused by poor material management and control, so it is necessary to strengthen the application of the data traceability function of blockchain. ⑧Information feedback: charitable donors can query completed projects, and the results achieved by specific projects, and are also able to receive feedback from beneficiaries.

#### 3) Information Platform

A specific information consultation platform and a special press officer are set up in the system. The information management personnel enter the system through a particular portal to manage all kinds of information. ①news release: it includes the timely release of news and activities related to disasters. The rapid spread of major disaster information can facilitate the understanding and participation of charitable donors; ②information release: the function is set as information release to foster help-seeking information release and charity donation information release. Through the charity donation platform, all parties as system users can release and handle accurate information in time; ③event notice: through system platform to track charity projects and timely issuing various activity notices, such as the convening, organization, and adjustment of major donation events; ④information modification: to modify and improve the information notice released in the system, and timely conduct deletion of overdue information; ⑤information exchange: to establish a particular discussion column and messaging hub, as well as the development of chat tools to promote all kinds of users to participate in discussions, messages, online meetings, etc., the exchange is based on the service system, to make more transparent the information between charity donors and beneficiaries, which is conducive to the maintenance and operation of charity projects.

**FIGURE 4.**
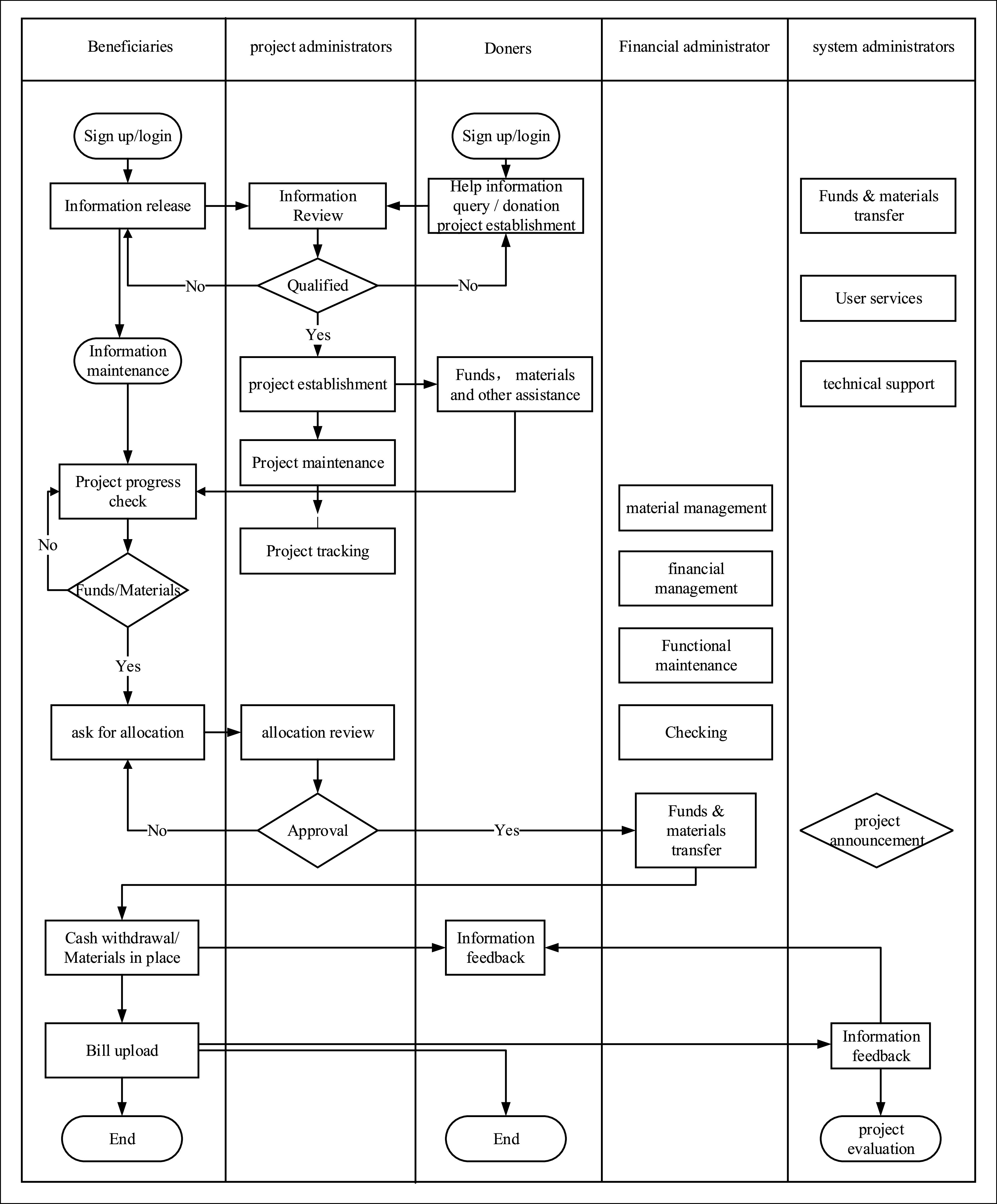
An operational module of the charity donation service system.

**FIGURE 5.**
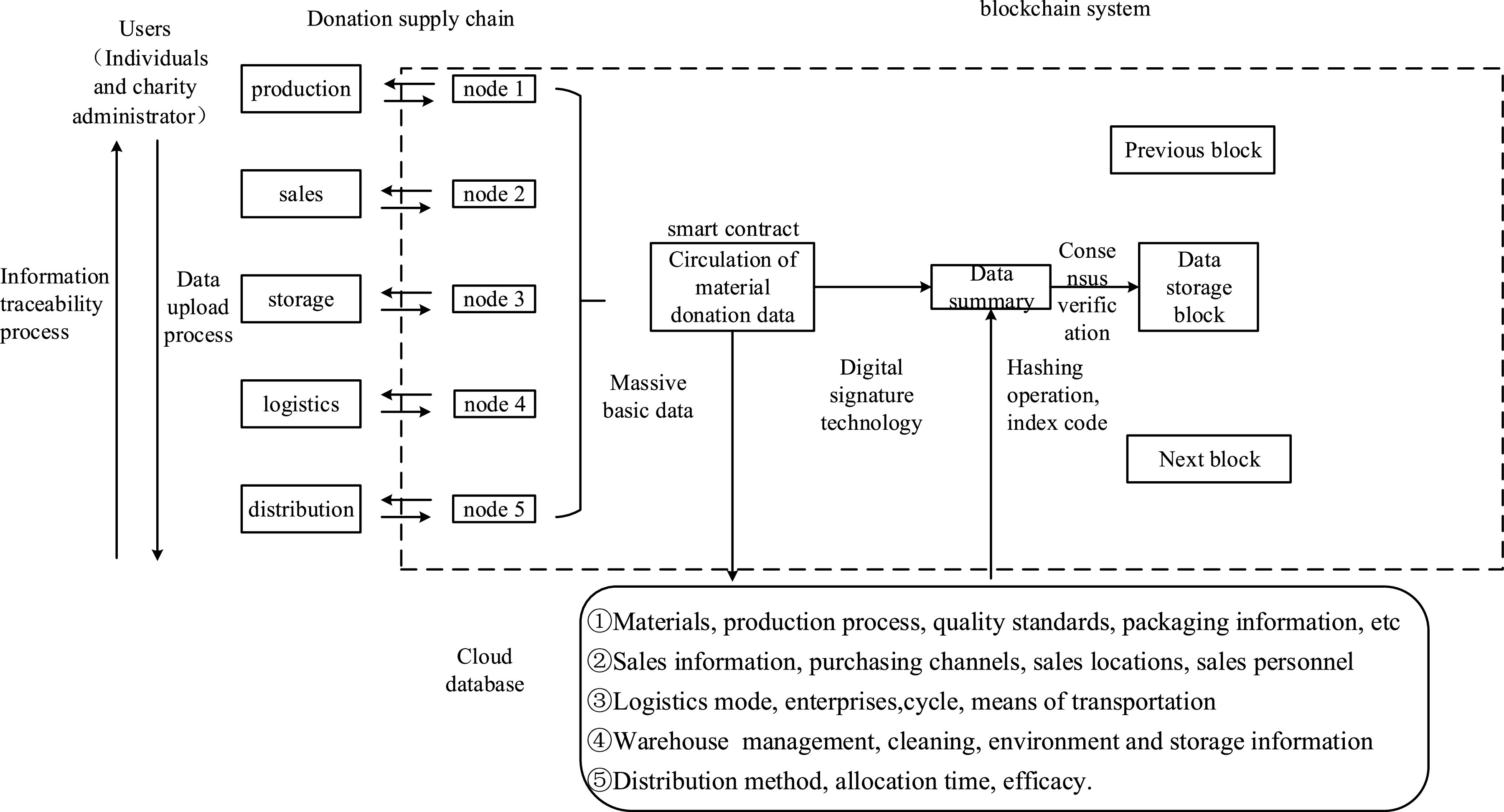
The Traceability model of charity donations.

#### 4) Project Management

Based on the service system, the whole process and comprehensive management of the initiated charity donation project is carried out to ensure the smooth operation of the charity donation service system. ①help-seeking audition: including the authenticity of identity and rationality of corresponding information on beneficiaries. According to the rescue standard set by the system, the manual method is adopted. Only those help-seeking users who meet the requirements and are reasonable can be uploaded to the system; ②project review: the project review initiated by charity donors can only be carried out after passing the assessment; ③project initiation: for specific and urgent charity donation matters, an individual project can be established, with the system manager serving as the initiator to promote the interaction between donors and beneficiaries; ④project maintenance: the system maintains the information of the projects initiated by the system and donors, and ensures that the project information is timely, transparent and effective, and overdue projects can be deleted in time; ⑤approval: review the fund and material allocation application, and only after the approval can it be allocated to the beneficiaries’ account for help; ⑥information announcement: the donors have the right to know specific information, so the report can ensure that users of all parties know on time to provide and receive timely assistance, and protect the donation willingness and rights of charitable donors.

#### 5) Financial Blockchain

The service system mainly guarantees the delivery of funds and materials donation services. The fund donation is carried out through the token system. The Ethereum account of charity donation is established, the token is issued, and the actual fund account is connected to ensure that the fund exchange between charity organizations and users is complete. The material management is reflected in the control of the logistics supply system, and the material procurement, transportation, deployment, and allocation are based on the blockchain, At the same time, the transaction data can be traced. ①Token issuance: the financial administrator uses the system platform based on the smart contract in Ethereum blockchain to develop a virtual token needed for a user account, and the exchange ratio is set to provide it to charitable donors for recharging and using. ②Material management: retrospective management of charity donation materials is established to manage the whole process from production or purchase to logistics system and finally allocate resources to beneficiaries, to attain the supervision and traceability of the entire process. ③Payment function: according to the authorization or permission of charitable donors, the beneficiaries can be given to token transfer payment or material permission. ④Token management: it issues the tokens required by system users, and conducts a financial audit on system tokens, to realize the business of token return, token reward, token forced recovery, etc.. ⑤Financial function maintenance: the system maintains the financial accounts of all users, mainly to ensure the safety of financial assets, timely control the accounts in case of abnormality, and timely delete necessary and false statements [56].

**FIGURE 6.**
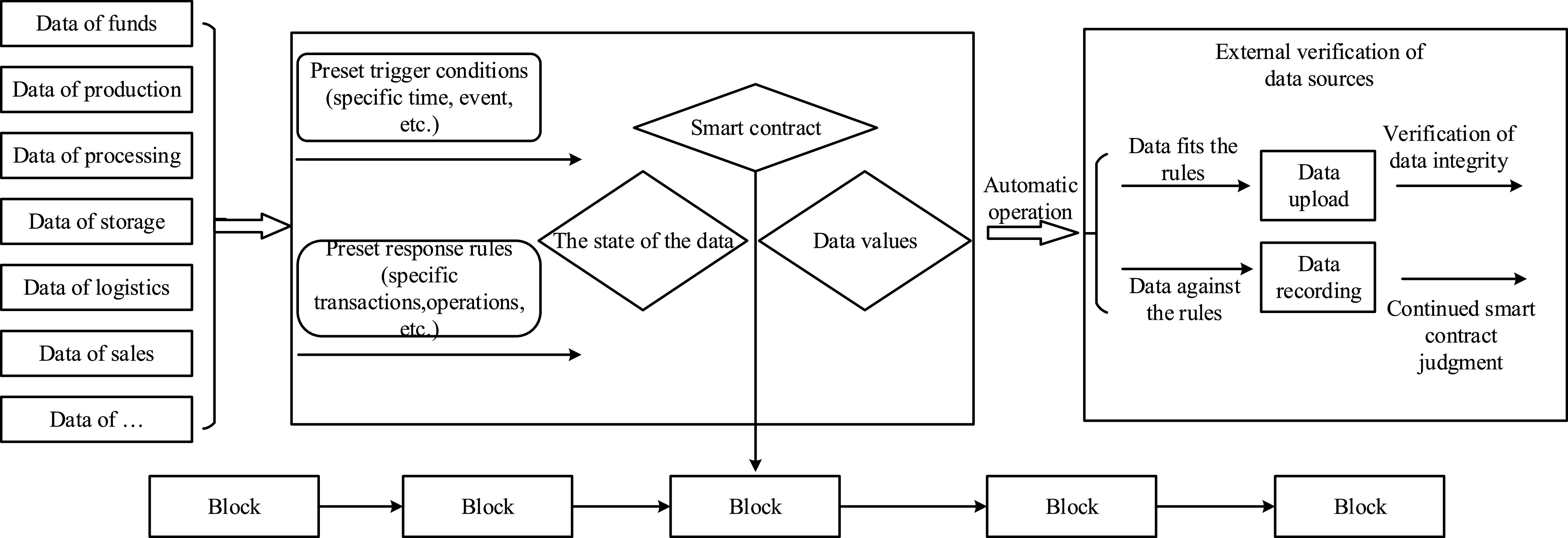
Operation process of smart contract.

#### 6) System Administrator

The system administrator mainly manages all accounts of the charity donation service system, grants authority to the administrator, classifies the system platform according to the administration, and monitors the operation of the system platform. ①User management is in charge of approving user registration, user information change, user authority management, user platform maintenance, and user complaint. ②Administrator account includes the establishment, query, modification, and deletion of administrator, authority setting, authority approval, and responsibility division. ③Technical support: for technical work, the customer service center is set up to organize technical personnel to provide online services and provide $7\times 24$ technical solutions immediately. ④Function development and maintenance: operators maintain and develop the service system, provide solutions for system improvement through user experience analysis, and provide suggestions for improving the operationality of the system, and also give feedback in the form of information record and information report.

## V. Implementation of Charity Donation Service System

Based on the combination of web and blockchain, the software architecture of the system is divided into three layers: front-end control layer, back-end control layer, and data service layer. In the front-end control layer, different interfaces are displayed to different users through the data service application platform. Users send business requests to the back-end control layer through the front-end controller. In the back-end control layer, the blockchain business controller and the web service controller respond to user needs and call services. This specific service module can not only accept blockchain services but also be completed by web services only according to the service requirements. In the data service layer, the transaction information on the blockchain can implement the smart contract, the local database data can be recalled and managed, the fund or material transactions of donors are stored on the blockchain, and the local database provides local data query.

Data traceability is the core of donation under the Covid-19 epidemic. Ethereum is a blockchain development platform, and its architecture is divided into seven layers: storage layer, data layer, network layer, protocol layer, consensus layer, contract layer, and application layer. The storage layer includes log data and metadata storage; the data layer includes data packaging, encryption, hash processing, block content digital signature, time stamp, Merkle tree, and chain structure data storage; the network layer carries out the information exchange and communication between the network nodes of the blockchain; the protocol layer ensures the protocol support required by each module in the Ethereum blockchain system when calling each other; the consensus layer provides proof of work (PoW) and proof of stake (PoS); through Ethereum virtual computer, the data and code of smart contract run on EVM; the last layer provides various applications for customers.

In funds management, the substitution of cryptocurrency for traditional funds can avoid transaction tampering. While the donation project is set up, the system issues tokens and completes the user’s rights of token exchange, transfer, and recovery by calling the qualified smart contract on the blockchain. The transaction information is stored in the Merkle tree of the blockchain, and the hash value of the transaction data is stored on the node. Due to the irreversibility and non-conflict of the hash value algorithm, the hash value of each transaction is unique. Firstly, the hash value corresponding to each transaction in the local database is queried, and the smart contract queries the corresponding transaction records and transaction data on the blockchain platform. This two-stage query ensures the accuracy of the data and solves the problem of authenticity and transparency.

**FIGURE 7.**
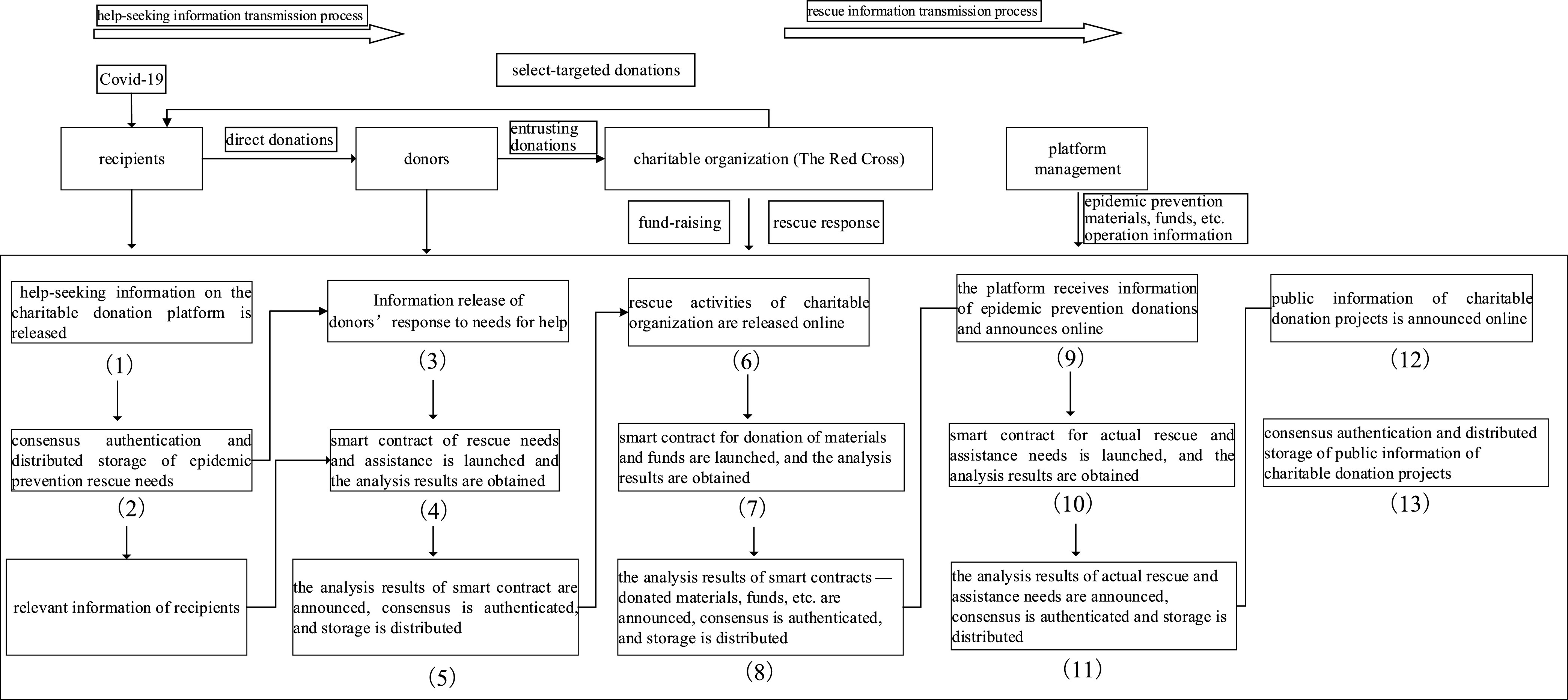
Operation mechanism of blockchain-based charity donation service system.

In materials management, the demand for prevention materials drives manufacturers, suppliers, distributors, retailers, logistics providers, charity management agencies, communities, and voluntary service agencies to form an integrated functional network chain of disaster relief materials supply. The core of disaster relief material supply blockchain management is the comprehensive control of goods flow, information flow, logistics, capital flow, etc. The business logic of charity donation material management is recorded on the blockchain in the form of a smart contract. As the typical role of the blockchain, the users access the blockchain network through an online platform or other distributed applications to obtain the information on the chain. Based on the alliance chain, the system strengthens the access mechanism and authority control. According to the function and storage node, the system gives different participation rights according to different roles. The operation of the whole chain can promote multi-party participation, data transparency, and traceability.

With the development of the Covid-19 epidemic, the amount of data increases sharply. It requires the cloud service platform and large node computer group to meet its operational requirements. The focus is to optimize the charity donation process, especially for the heavily affected areas such as Wuhan. Information within the system and external related information, such as donation demand, fund and material management policies, guidance from national disease control agencies, etc., are transmitted and flowed throughout the network through the P2P communication system of the blockchain system, so as to facilitate the coordinated control within the system.

The operation of the service system will inevitably bring a large number of complicated multi-source heterogeneous data. By using smart contract, we make the data consistent on each node. Only consistent data can be uploaded. At the same time, in order to avoid different formats of data information from different users, the data needs to be judged by smart contract, and then verified by digital signature technology. The digital signature technology is mainly to check whether the data stored in the cloud database is effective and reliable. Each link of the service system should formulate the agreement terms on smart contract according to the actual requirements. In the data processing, the data is judged according to the preset trigger conditions to ensure the integrity verification step by step. Under the Covid-19 outbreak, the information transmission between each link can be carried out according to the existing smart contract. The system has the characteristics of reliable traceability. From the release of epidemic information, to material procurement and supply, token issuance and exchange, the system connects the information exchange between users, which can improve the transparency of current charity activities, and improve the credibility of current charity and public welfare. The system has excellent security and traceability, which has a certain significance and value for the charity donation activities.

## VI. Conclusion

The Covid-19 epidemic has brought new challenges to the establishment and operation of charity donation service system. The introduction of blockchain technology has thus emerged to address cross-regional and cross-domain charity donations issues. The blockchain technology can be used for protecting the data security, defining access policies, ensuring the transparency of donations, and traceability of donation behaviors. It is an emergency response to specific regional disaster in the wake of the changing Covid-19 status. The ultimate goal of our research is to fulfill that blockchain-supported solution taps into the integration of traditional web service and blockchain technology, speeds up the system development and then responds to the needs of users in a timely fashion. The Covid-19 situation has also given rise to a large demand of funds and materials. As the transaction voucher of the system, cryptocurrency can thus ensure the security of transaction records, identity data, and relevant details. It is powered to monitor the process of capital flow and improve the functional network chain of relief materials. We also emphasize the comprehensive management and handling capability of material supply, and establish a charity donation service system with sustained innovation in framework, technology, and operation. Blockchain has receiving more attention in the charity donation system in sharing donation data, in managing information among donors and beneficiaries, in contract management among charitable organizations and enterprises, and its application in dealing with the Covid-19-centered donations are growing increasingly day to day.
